# Supplementary material for: Zfra Inhibits the TRAPPC6AΔ-Initiated Pathway of Neurodegeneration
Source: Int J Mol Sci. 2022 Nov 22;23(23):14510. doi: 10.3390/ijms232314510 (PMC9739312; doi:10.3390/ijms232314510)
Supplement: Supplementary file 1 [file ijms-23-14510-s001.zip › ijms-1941526-supplementary.pdf]

Supplementary Information

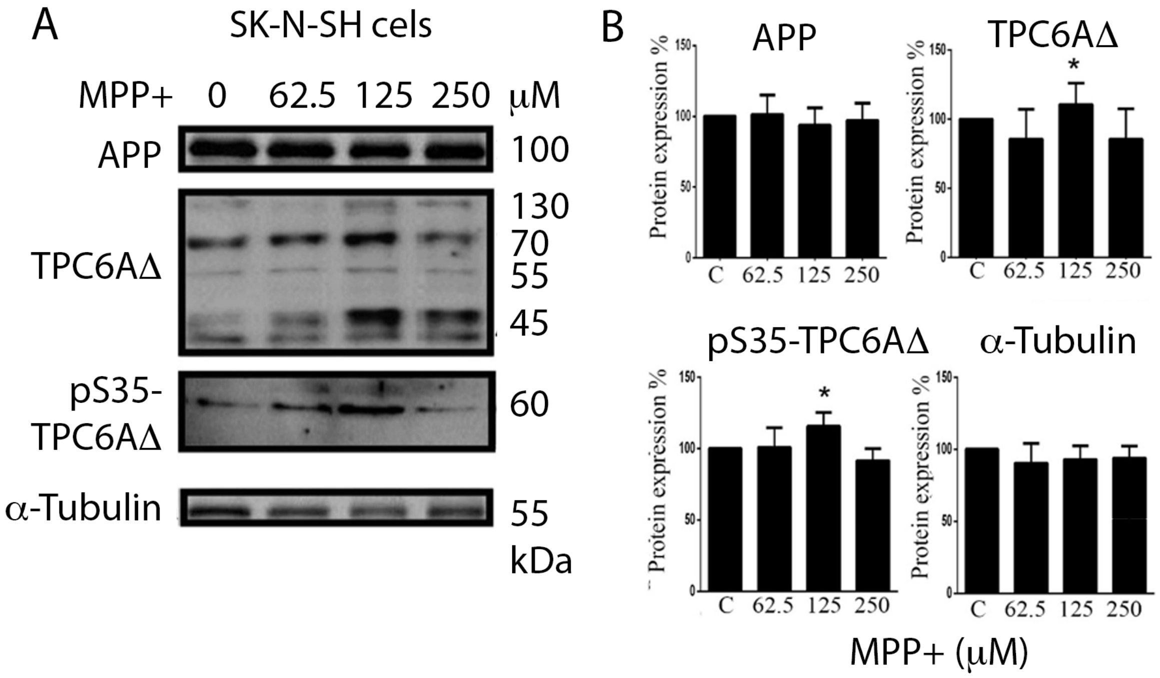

Figure S1. MPP+ induces the expression of TPC6AΔ in a dose-dependent manner. (**A**,**B**) SK-N-SH cells were treated with various concentrations of MPP+ for 24 hours. The expression levels of APP, TPC6AΔ, pS35-TPC6AΔ, and *α*-tubulin were determined by immunoblotting and quantified (Bonferroni's Student T test: * $p < 0.05$, ** $p < 0.01$ vs. respective control group. n = 3). Gels were run under non-reducing conditions.

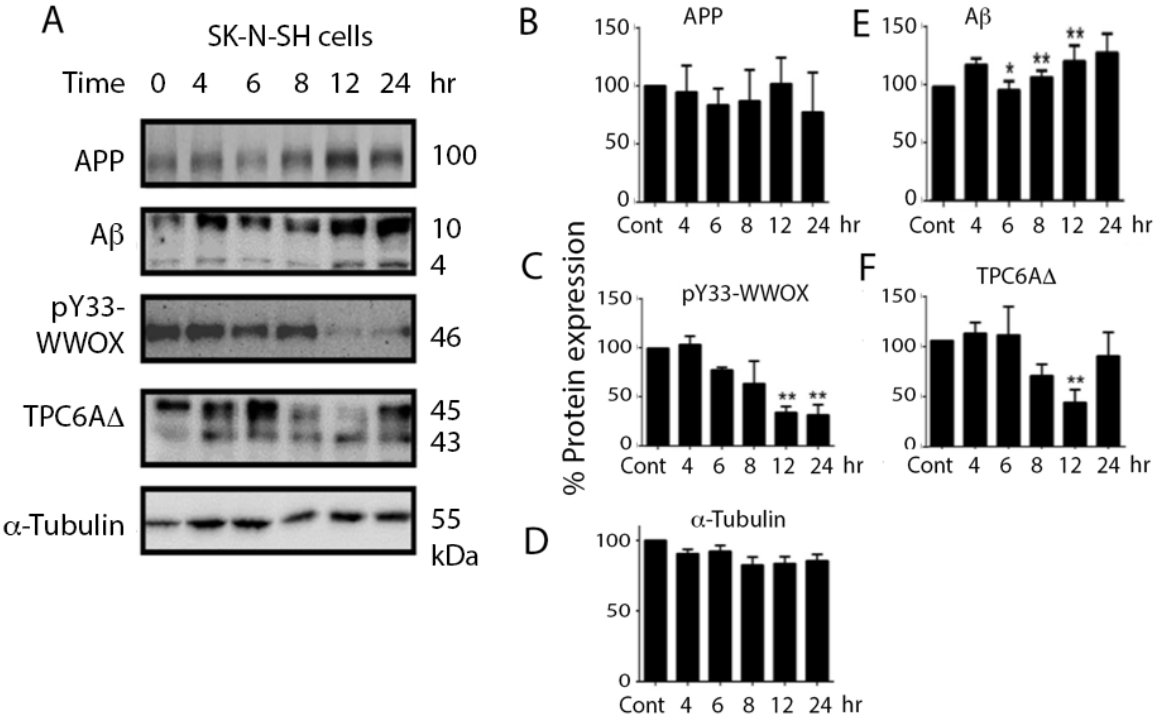

Figure S2. Time-related induction of the expression of TPC6AΔ by MPP+. (A-F) SK-N-SH cells were treated with MPP+ (125 μM) for indicated times. The expression levels of APP, Aβ, pY33-WWOX, TPC6AΔ, pS35 and *α*-tubulin were determined by immunoblotting (**A**) and quantified (**B-F**) (Bonferroni's student T test: * $p < 0.05$, ** $p < 0.01$ vs. respective control group. n = 3).

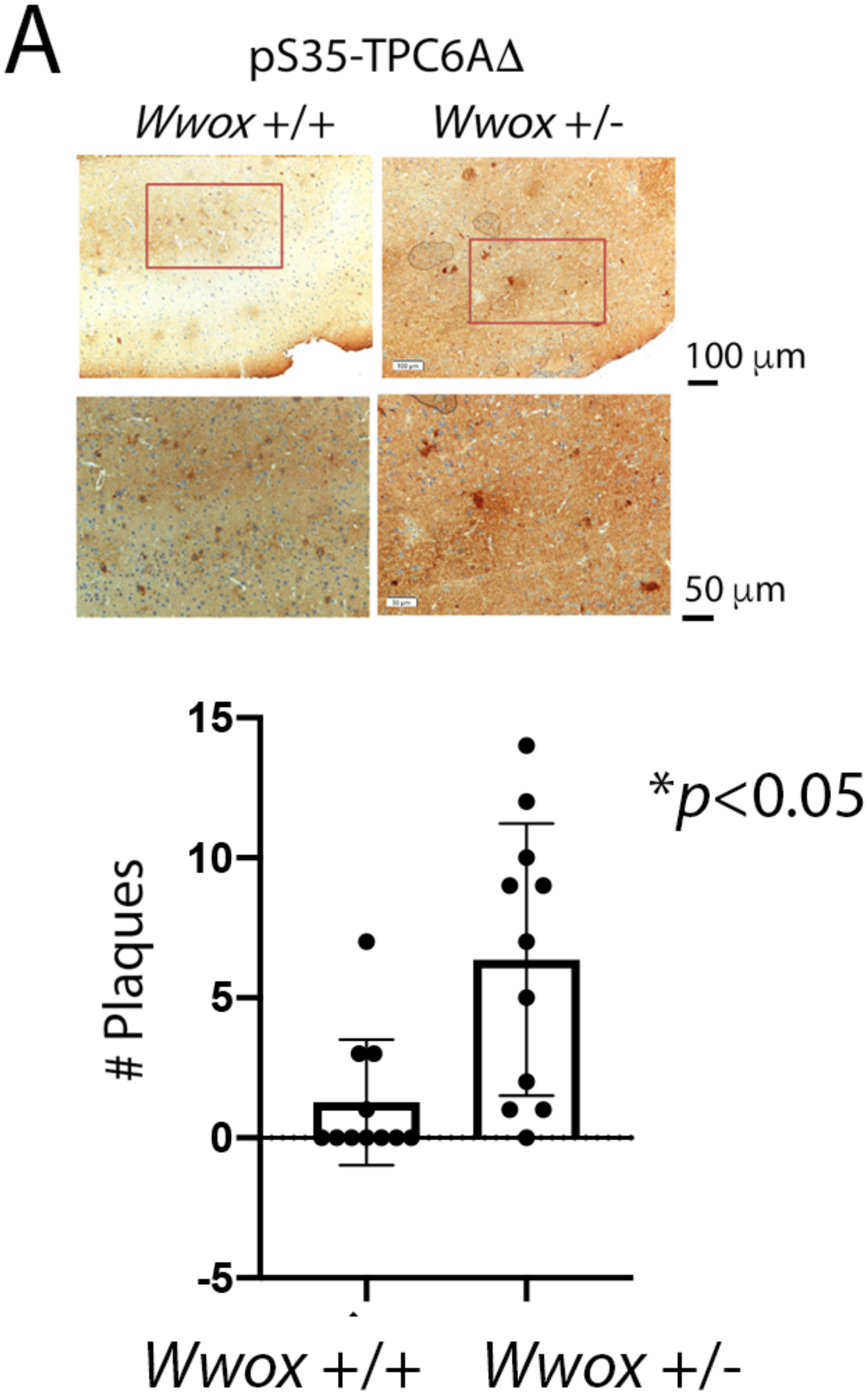
A
pS35-TPC6AΔ
Wwox +/+
Wwox +/-
100 μm
50 μm
15
10
5
0
-5
Plaques
*p<0.05
Wwox +/+
Wwox +/-

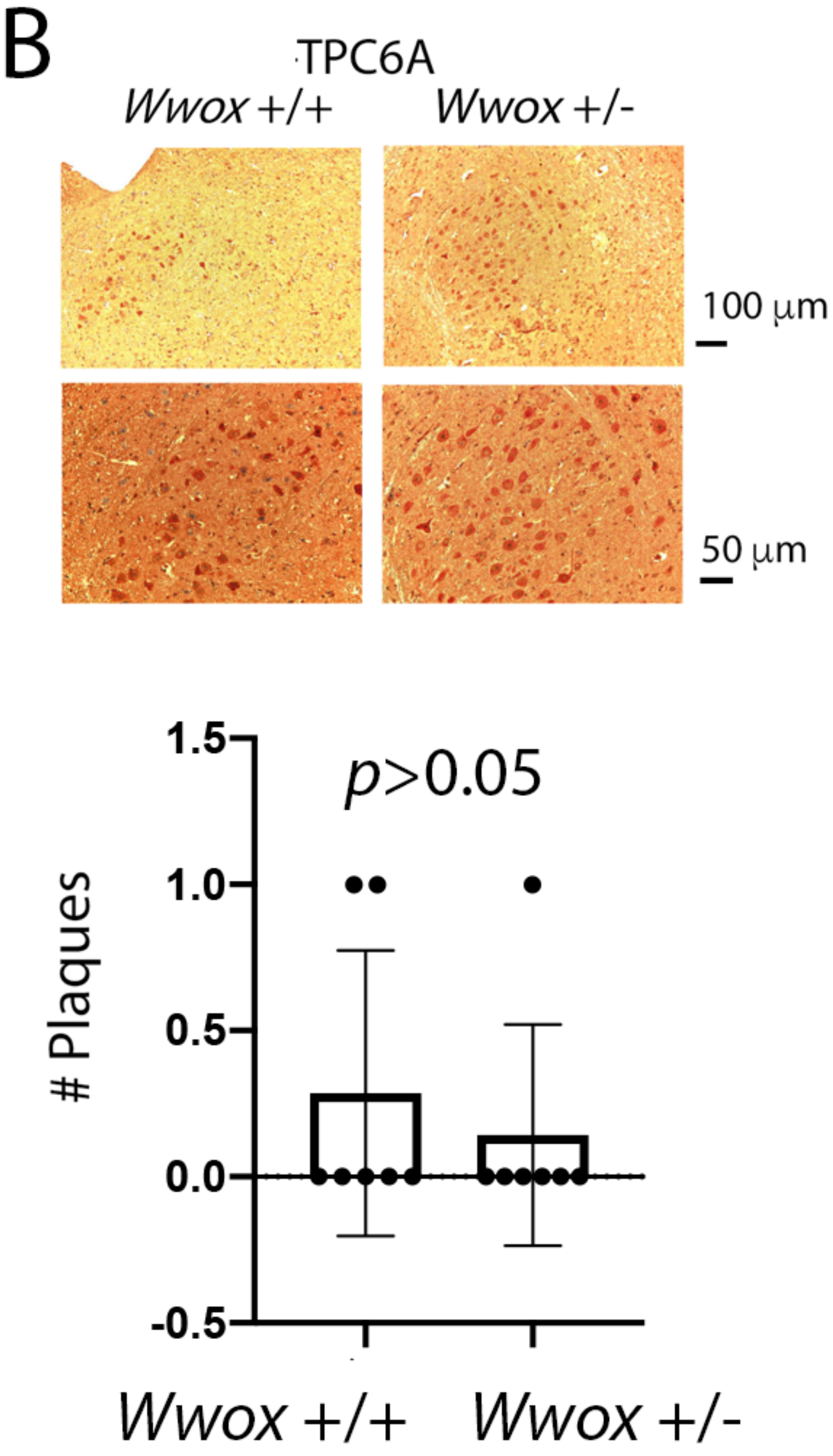
B
TPC6A
Wwox +/+
Wwox +/-
100 μm
50 μm
1.5
1.0
0.5
0.0
-0.5
p>0.05
Plaques
Wwox +/+
Wwox +/-

C

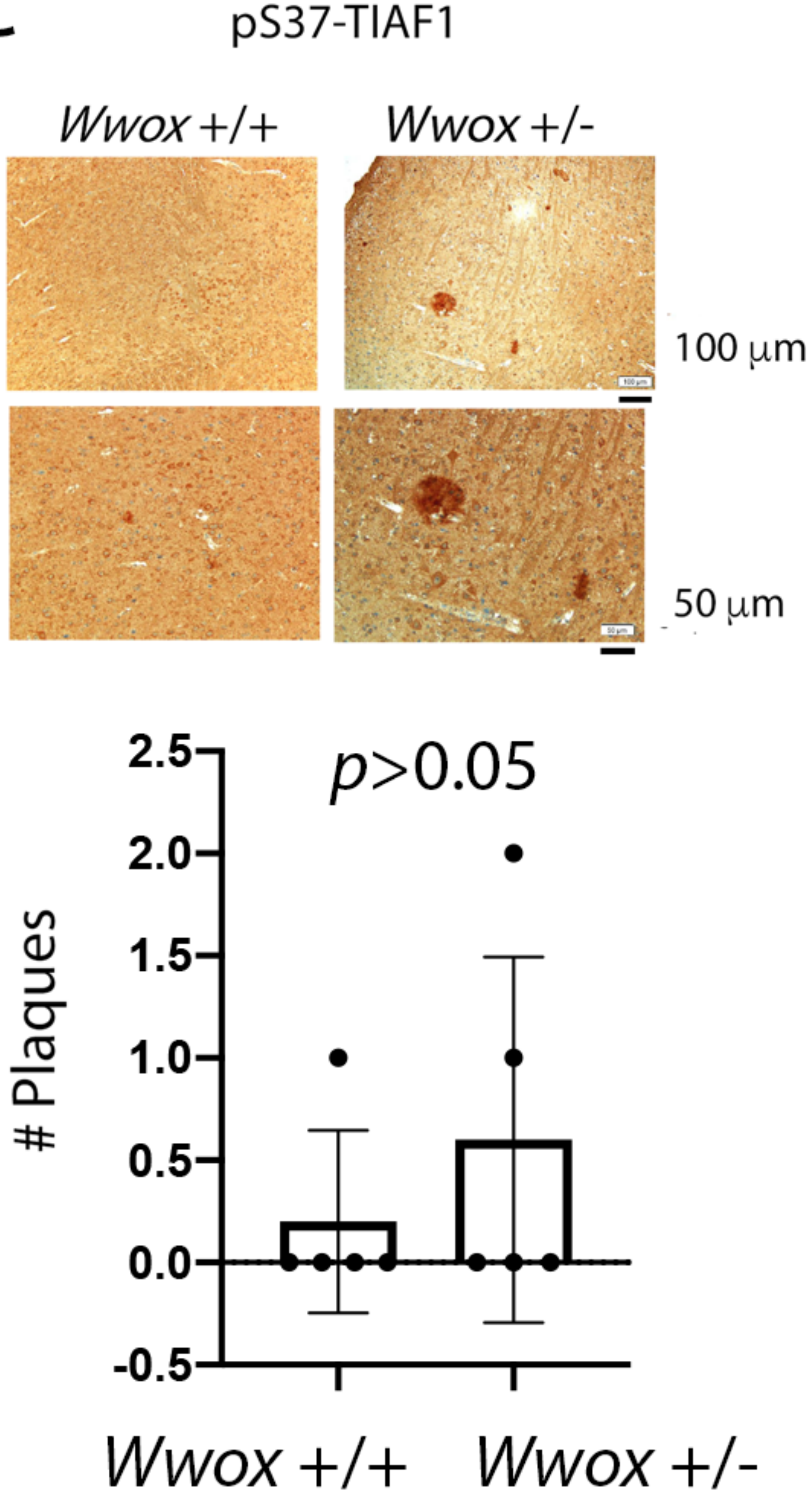

pS37-TIAF1
Wwox +/+
Wwox +/-
100 μm
50 μm
p>0.05
Plaques
2.5
2.0
1.5
1.0
0.5
0.0
-0.5
Wwox +/+
Wwox +/-

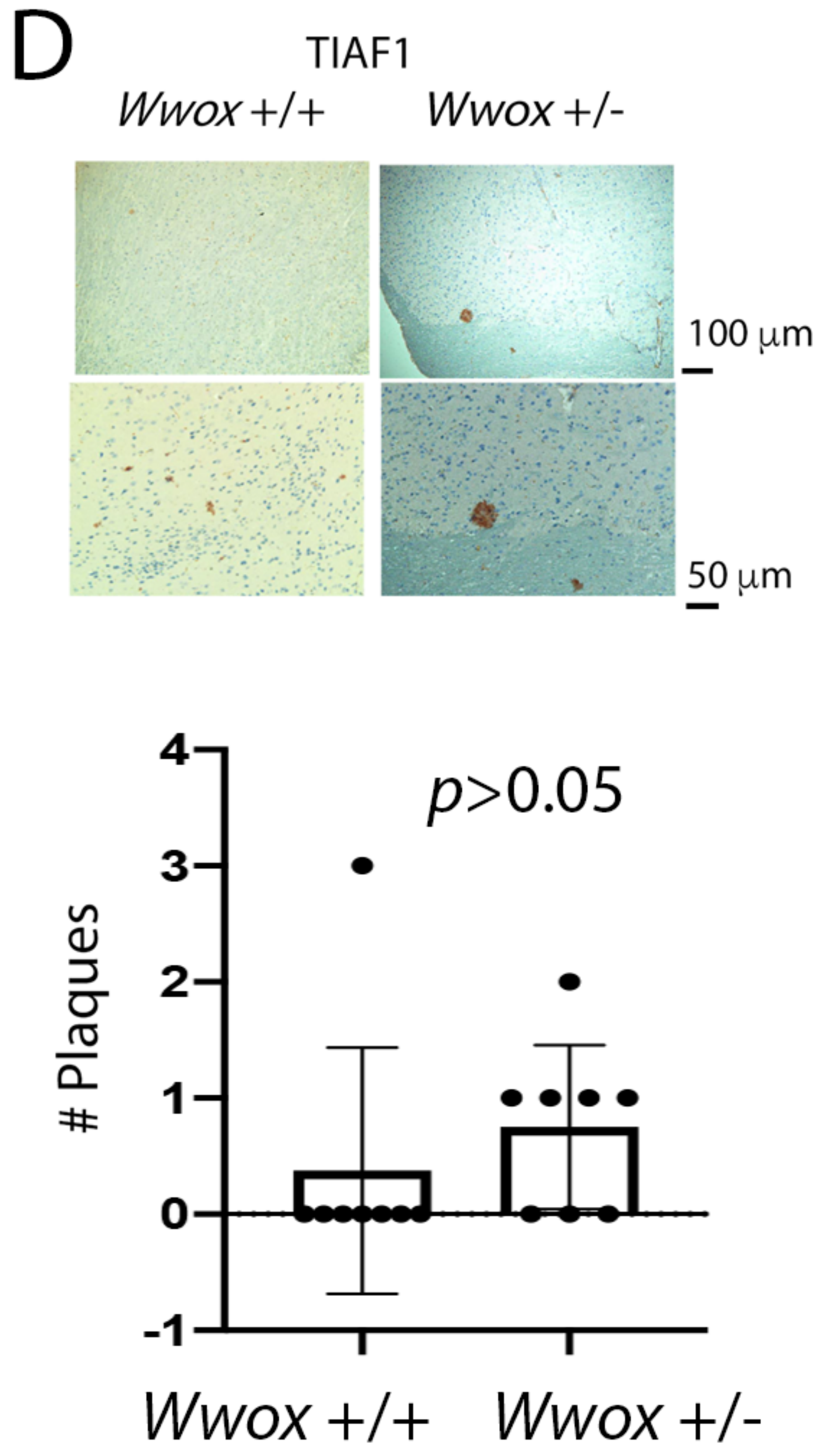
D
TIAF1
Wwox +/+
Wwox +/-
100 μm
50 μm
4
3
2
1
0
-1
Plaques
p>0.05
Wwox +/+
Wwox +/-

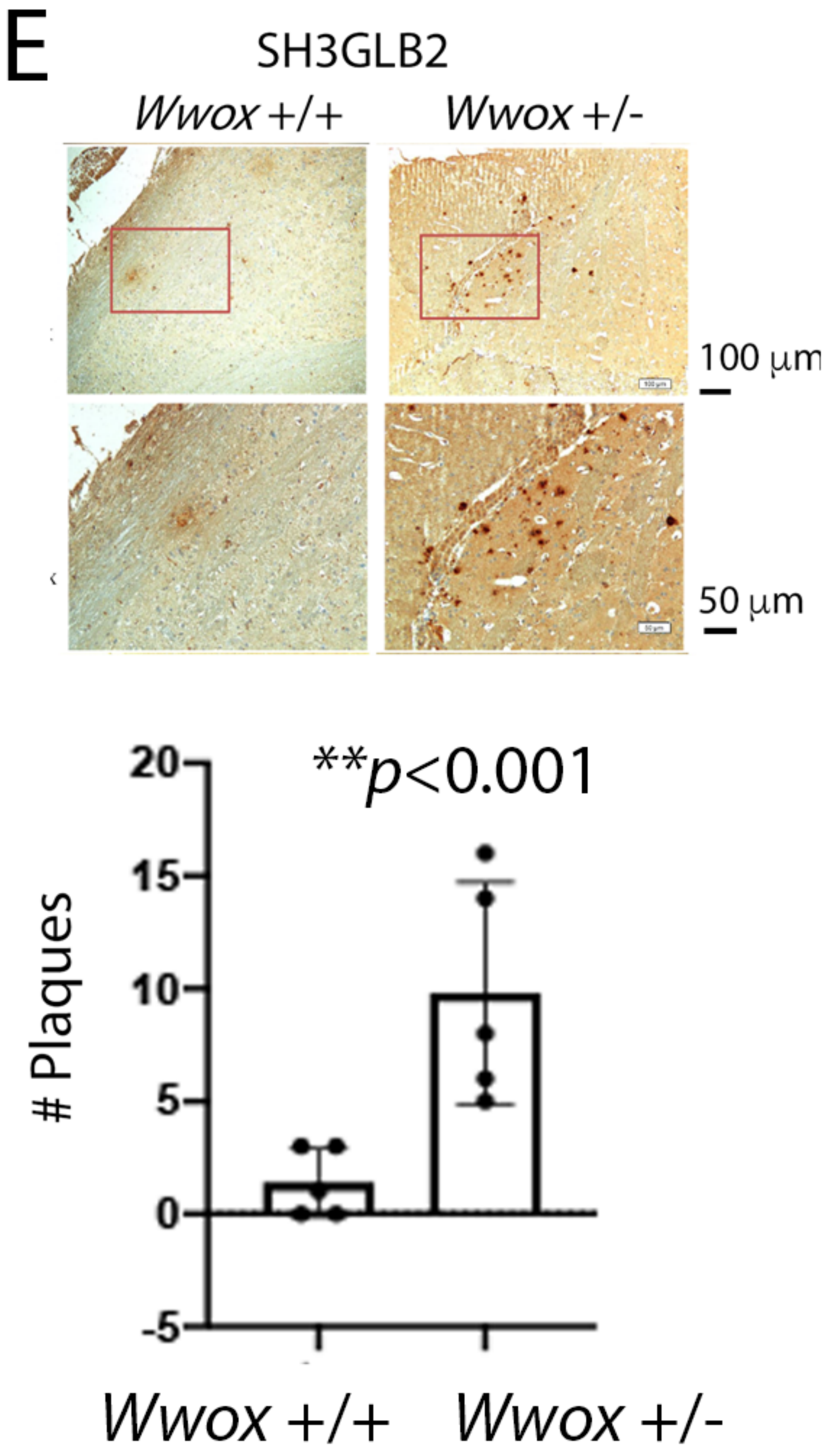

E
SH3GLB2
Wwox +/+
Wwox +/-
100 μm
50 μm
**p<0.001
Plaques
20
15
10
5
0
-5
Wwox +/+
Wwox +/-

F

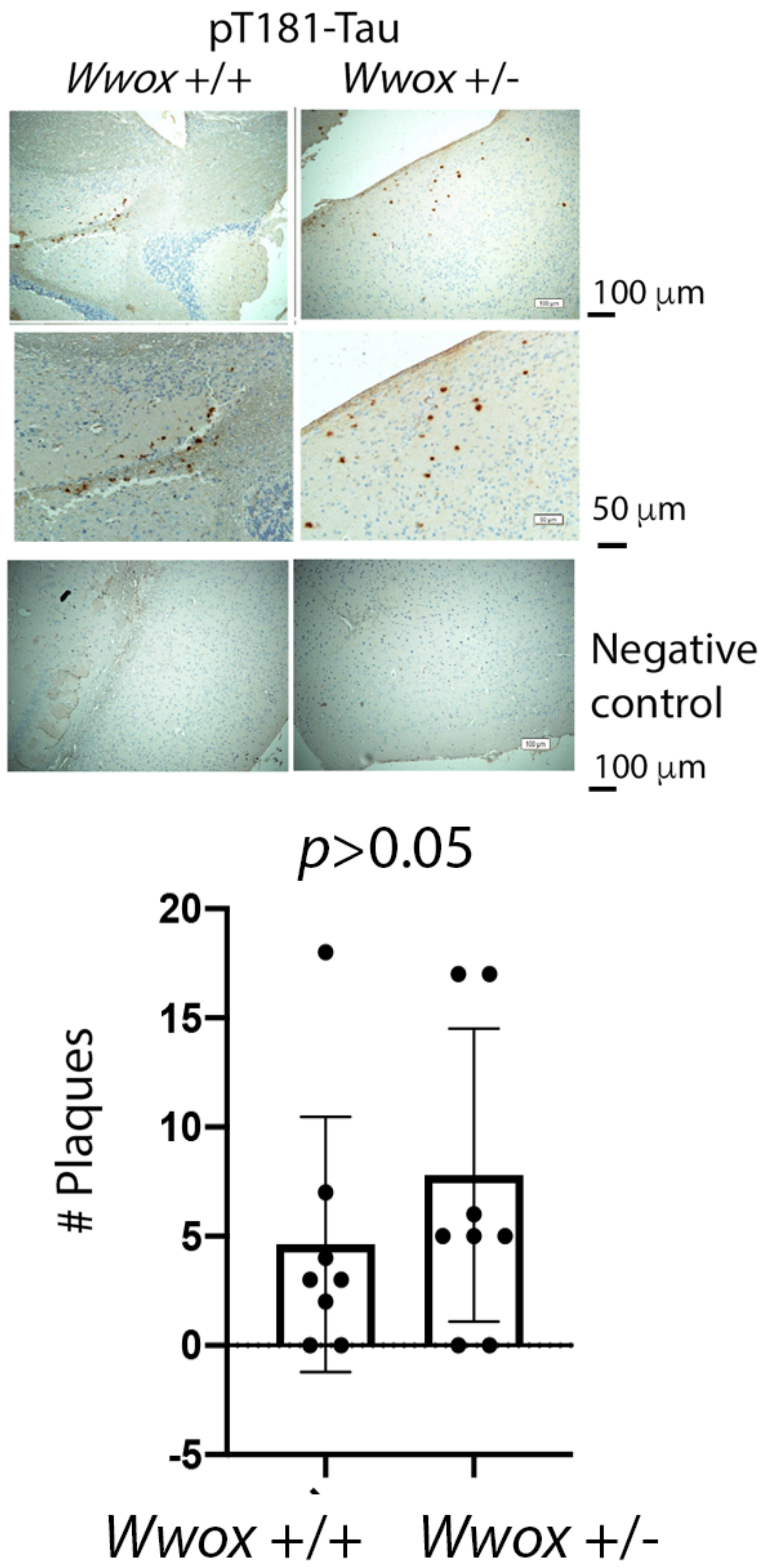
pT181-Tau
Wwox +/+
Wwox +/-
100 µm
50 µm
Negative
control
100 µm
p>0.05
20
15
10
5
0
-5
Plaques
Wwox +/+
Wwox +/-

Figure S3. Digitally enlarged Figure 6 showing the significantly increased pS35-TPC6AΔ and SH3GLB2 aggregates in the brain cortex of 11-month-old heterozygous *Wwox* mice. (A-F) By immunohistochemistry, protein expression of pS35-TPC6AΔ (**A**), TPC6A (wild type) (**B**), pS37-TIAF1 (**C**), TIAF1 (**D**), SH3GLB2 (**E**), and pT181-Tau (**F**) was examined in the brain cortex of 11-month-old heterozygous *Wwox* mice and age-matched wild type mice. The extent of protein aggregation was assessed (n=5 to 10; Student's t test).

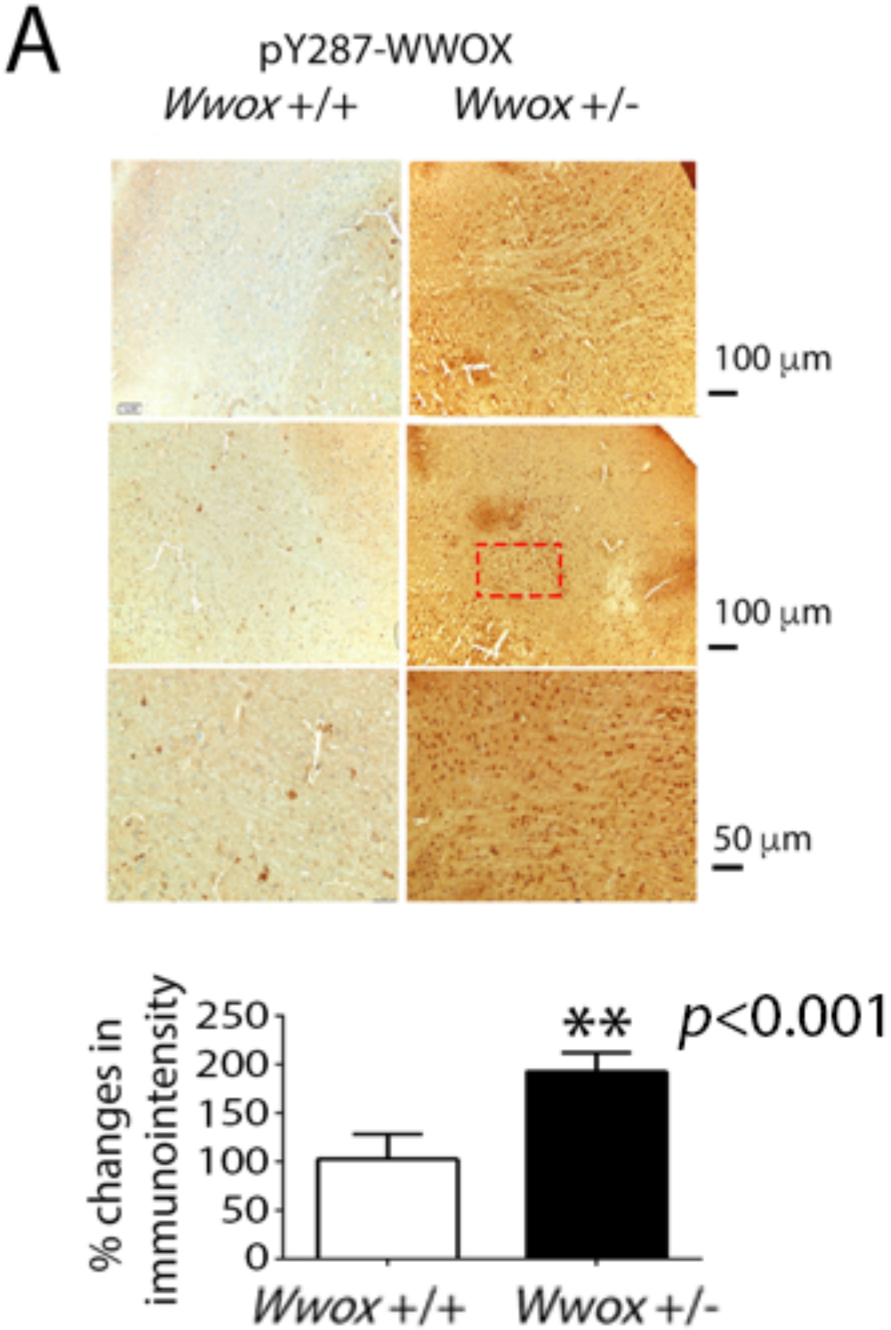
A
pY287-WWOX
Wwox +/+
Wwox +/-
100 μm
100 μm
50 μm
% changes in immunointensity
250
200
150
100
50
0
**
p<0.001
Wwox +/+
Wwox +/-

B

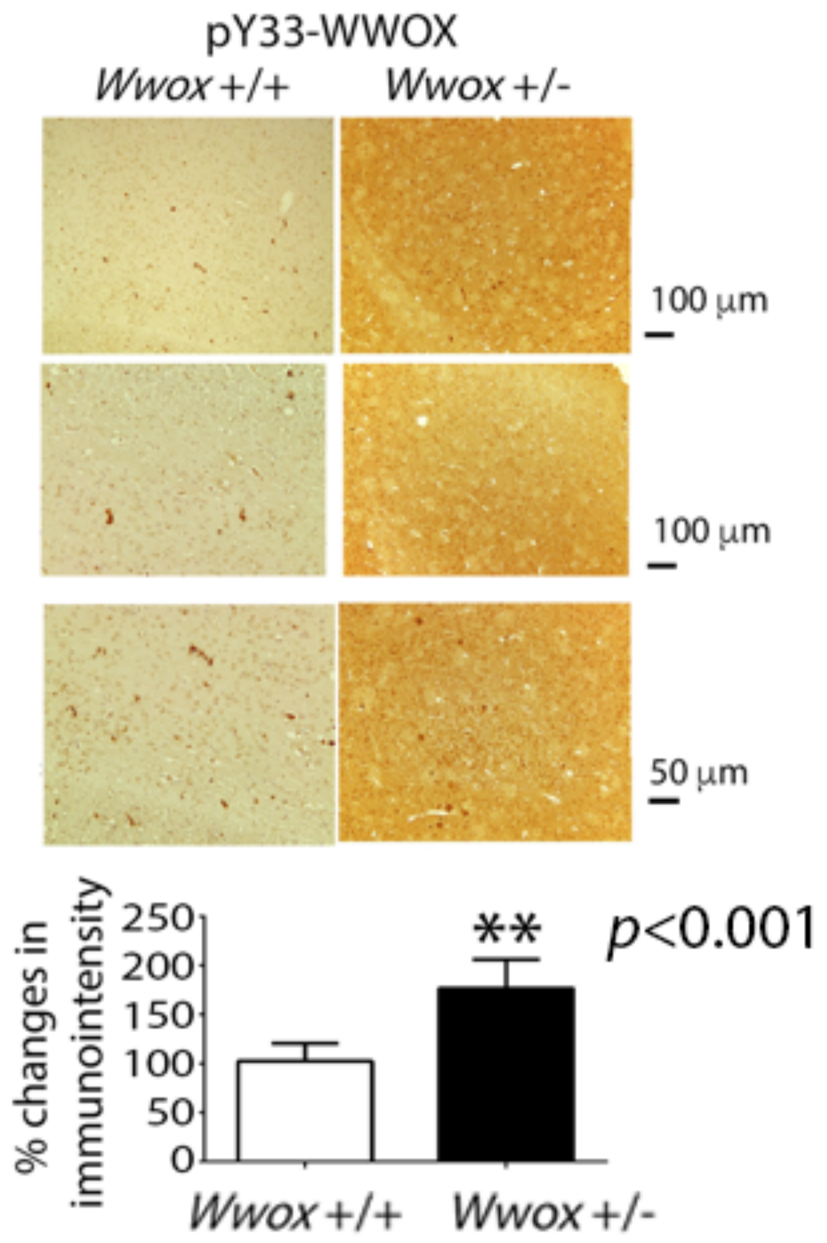
pY33-WWOX
Wwox +/+
Wwox +/-
100 μm
100 μm
50 μm
% changes in immunointensity
250
200
150
100
50
0
**
p<0.001
Wwox +/+
Wwox +/-

C

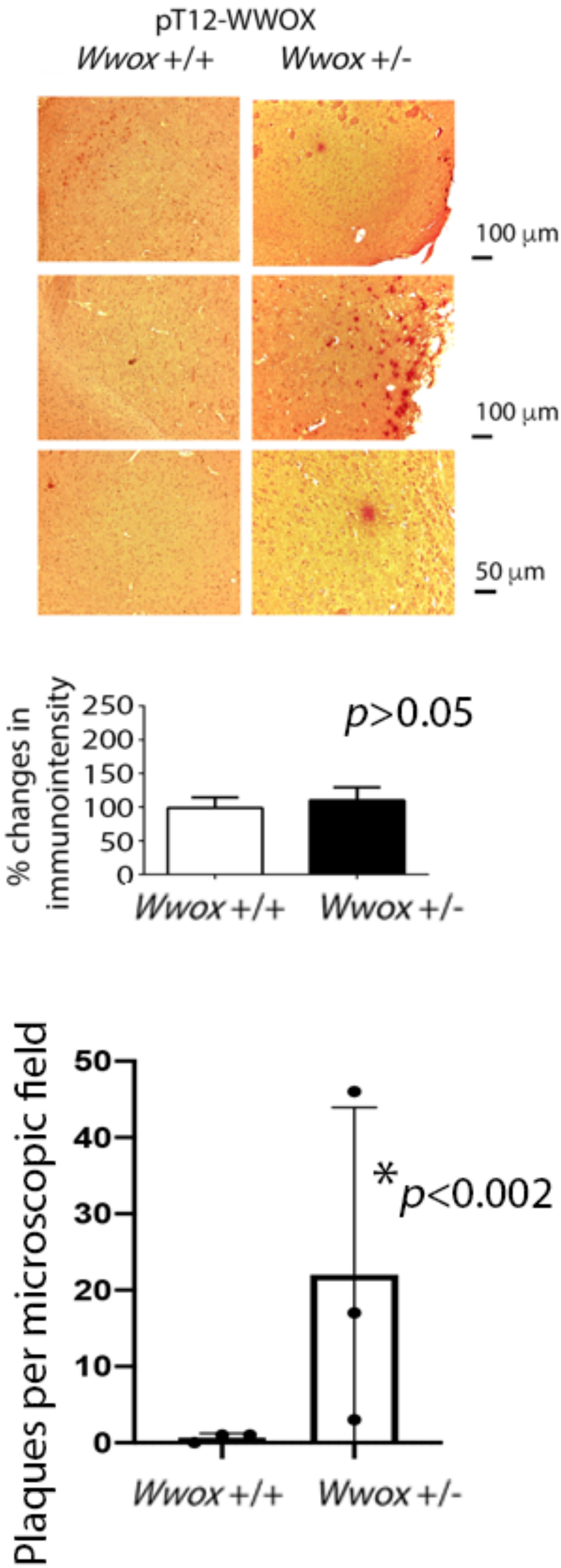
pT12-WWOX
Wwox +/+
Wwox +/-
100 µm
100 µm
50 µm
% changes in immunointensity
250
200
150
100
50
0
p>0.05
Wwox +/+
Wwox +/-
Plaques per microscopic field
50
40
30
20
10
0
*p<0.002
Wwox +/+
Wwox +/-

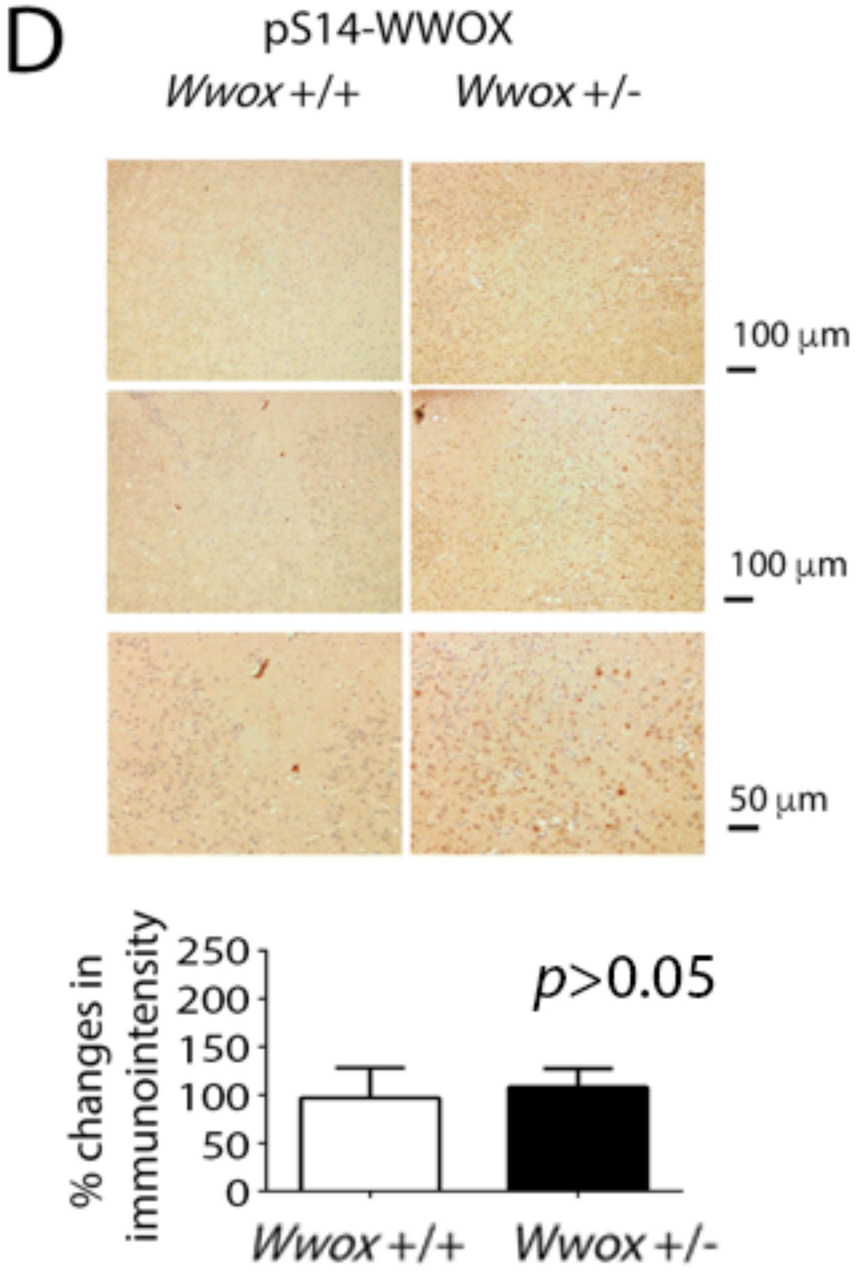
D
pS14-WWOX
Wwox +/+
Wwox +/-
100 μm
100 μm
50 μm
% changes in immunointensity
250
200
150
100
50
0
p>0.05
Wwox +/+
Wwox +/-

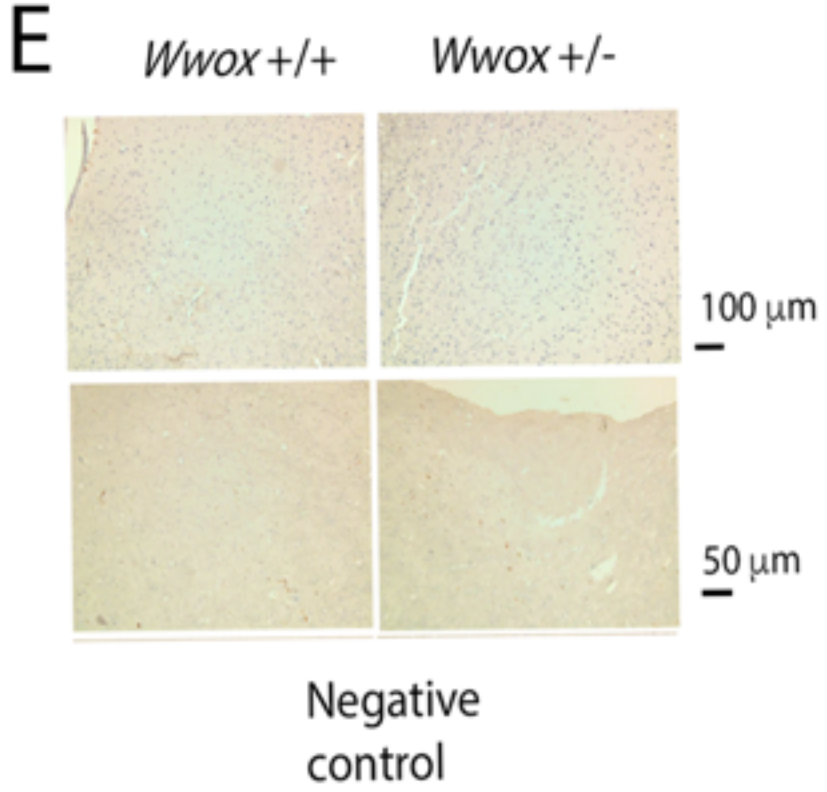

Figure S4. Digitally enlarged Figure 7 showing identification of pT12-WWOX as aggregates in the brain cortex of 11-month-old heterozygous *Wwox* mice. (A-E) Compared to the wild type mice, increased immunointensity of staining is shown in the cortex of heterozygous *Wwox* mice using antibody against pY287-WWOX (**A**) and pY33-WWOX (**B**), respectively. Presence of pT12-WWOX aggregates (**C**), but not pS14-WWOX (**D**), is shown in the cortex of heterozygous *Wwox* mice. No pT12-WWOX aggregates were found in the wild type mice. The bar graphs show mean ± standard deviation (n=5; ***p*<0.001, Student's t tests). The number of plaques per microscopic field (100x magnification) is shown for pT12-WWOX (n=3) (**C**). No plaques are shown with pS14-, pY33-, and pY287-WWOX. (**E**) Negative controls without staining with primary antibodies are shown.

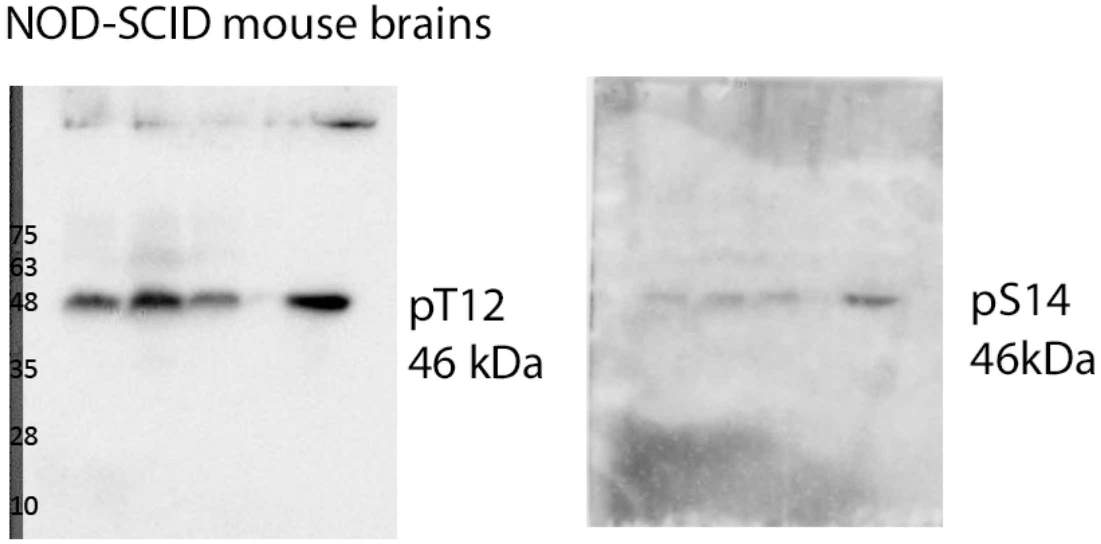

Figure S5. Antibody against pT12-WWOX. The whole brain extracts from 5 NOD-SCID mice were prepared and subjected to reducing SDS-PAGE. This was intended to test the quality of our newly generated antibody. We had made antibody against pS14-WWOX [19].
